# The effect of occupational exposure to organic dust on lung function parameters among African industrial workers: a systematic review and meta-analysis

**DOI:** 10.3389/fpubh.2024.1424315

**Published:** 2024-11-01

**Authors:** Zemachu Ashuro, Berhanu Gidisa Debela, Chala Daba, Habtamu Endashaw Hareru, Samson Wakuma Abaya, Anthony L. Byrne

**Affiliations:** ^1^School of Public Health, College of Health Science and Medicine, Dilla University, Dilla, Ethiopia; ^2^Department of Environmental Health, College of Medicine and Health Sciences, Wollo University, Dessie, Ethiopia; ^3^Department of Preventive Medicine, School of Public Health, College of Health Sciences, Addis Ababa University, Addis Ababa, Ethiopia; ^4^St Vincent's Hospital, University of New South Wales, Sydney, NSW, Australia

**Keywords:** Africa, exposure, spirometry, organic dust, lung function

## Abstract

**Introduction:**

Inadequate ventilation and improper use of personal protective equipment are often observed in many occupational settings with a high risk of dust and other fine particle exposure. Workers who are exposed to dust at work may suffer from respiratory difficulties. Previous systematic reviews on organic dust exposure and its association with respiratory health outcomes did not provide a comprehensive assessment. Therefore, the objective of this systematic review and meta-analysis was to summarize the reported effects of organic dust exposure on lung function parameters among African industrial workers.

**Methods:**

A compressive literature search was conducted in PubMed, MEDLINE, Google Scholar, Embase, the Web of Science, African Journals Online, and ScienceDirect databases to identify relevant studies for the review. The Newcastle–Ottawa Scale (NOS) was used to assess the quality of the included studies. The lung function indices including forced vital capacity (FVC), forced expiratory volume in the first second (FEV_1_), the FEV_1_/FVC ratio, and peak expiratory flow rate (PEFR) were obtained from primary studies and analyzed using STATA version 17. The *I*^2^ test was used to assess the heterogeneity of studies. We used a random-effects model to estimate the pooled standard mean difference in lung function indices between organic dust-exposed and non-exposed industrial workers. To analyze publication bias, funnel plots and Egger’s test were applied.

**Results:**

In this systematic review and meta-analysis, 32 studies involving 7,085 participants were included from 13,529 identified studies. The estimated mean differences with 95% confidence intervals were as follows: −0.53 [−0.83 to −0.36] L for FVC, −0.60 [−0.77 to −0.43] L for FEV_1_, −0.43 [−0.57, −0.29] L for FEV_1_/FVC, and −0.69 [−0.88 to −0.50] L/min for PEFR.

**Conclusion:**

This systematic review and meta-analysis revealed that the lung function indices, such as FVC, FEV_1_, FEV_1_/FVC, and PEFR, were statistically significantly lower among organic dust-exposed industrial workers compared to non-exposed industrial workers. Therefore, effective dust control measures should be implemented to protect workers from exposure to organic dust.

**Systematic Review Registration:**

https://www.crd.york.ac.uk/prospero/display_record.php?ID=CRD42024527139.

## Introduction

Dust is defined as tiny dry particles in the air that are produced during manufacturing or production processes, such as cutting, drilling, grinding, or sawing (1). Dust generated in the workplace is divided into two types: organic dust and inorganic dust. Inorganic dust originates from non-living materials, such as stones, chemicals, and metals, generated during various industrial manufacturing and production processes. Workers are exposed to inorganic dust, such as cement, coal, asbestos, metal, concrete, stone, and sand, due to poor occupational health and safety practices (2–5). On the other hand, organic dust originates from living materials and includes dust from textiles, wood, poultry, leather, grain, and wheat, as well as spores from fungi and bacteria produced by industries during manufacturing or production processes (5, 6).

Dust-related adverse health effects are primarily determined by particle size, with microscopic and ultra-fine particles capable of penetrating deeply into the human respiratory system and causing serious health problems (7–10). The most serious health consequences of organic dust exposure among workers in work environments include lung, throat, and nose cancers, as well as other lung diseases known as chronic obstructive pulmonary disease (COPD), which includes chronic bronchitis and emphysema (11–14).

Organic dust exposure in the workplace can be hazardous to the respiratory health of industrial workers. Inhaling organic dust, such as cotton dust, wood dust, flour dust, paper dust, grain dust, animal confinement dust, or compost dust, can cause inflammatory responses in the respiratory system and airway obstruction (15, 16). Several studies have found that exposure to organic dust impairs lung function and induces respiratory symptoms (17–20). Exposure to endotoxins primarily causes respiratory consequences, such as reduced lung function and an increased prevalence of chronic bronchitis and asthma (21, 22).

Several studies have investigated the relationship between work-related organic dust exposure and reduction in lung function parameters across various industries, such as textiles (particularly cotton), paper, wood working, agriculture, flour milling, and grain processing, among industrial workers (23–27). According to research findings, organic dust exposure causes a decline or reduction in lung function parameters such as forced vital capacity (FVC), forced expiratory volume in the first second (FEV_1_), and the ratio of the two volumes (FEV_1_/FVC) among industrial workers exposed to dust in occupational settings due to inadequate ventilation and improper use of personal protective equipment (23, 25, 26, 28–42).

Several studies have been conducted in Africa to evaluate the levels of lung function decline; however, the outcomes vary significantly. Furthermore, the lack of a national occupational respiratory disease recording and reporting system results in varying estimates of respiratory disease prevalence and the inability to show the magnitude of lung function reduction, further limiting prevention efforts in the industry. Given the disparities in findings of previous studies and the lack of a national occupational respiratory disease recording and reporting system, we conducted a systematic review and meta-analysis to determine the overall impact of organic dust exposure on lung function among African industrial workers. This systematic review and meta-analysis aimed to help in the development of appropriate occupational safety and health policies and programs for implementing effective interventions. Furthermore, the findings of this study will provide valuable information to the Ministry of Health and the International Labour Organization (ILO) in promoting the health and wellbeing of industrial workers.

### Review question

What are the overall estimates of lung function parameters—FVC (liters), FEV1 (liters), FEV1/FVC ratio, and peak expiratory flow rate (PEFR) (liters/min)—among African industrial workers exposed to organic dust?

## Materials and methods

### Search strategy

This systematic review and meta-analysis protocol was registered in the International Prospective Register of Systematic Reviews (PROSPERO) and can be accessed at https://www.crd.york.ac.uk/prospero/display_record.php?ID=CRD42024527139. The literature search was carried out in accordance with the Preferred Reporting Items for Systematic Reviews and Meta-Analyses (PRISMA) criteria (43, 44).

We conducted a compressive literature search in various international databases, including PubMed, MEDLINE, Embase, the Web of Science, African Journals Online, and ScienceDirect, to obtain relevant published primary studies for this systemic review and meta-analysis. In addition, we searched gray literature and university databases for unpublished studies to identify all relevant articles.

An extensive search of the databases was conducted using the following keywords or MeSH terms: “lung” [MeSH Terms] OR “pulmonary” [All Fields] AND “respiratory function tests” [MeSH Terms] OR “lung function” [All Fields] AND “lung function reductions” [All Fields] AND “organic agriculture” [MeSH Terms] OR “organic” [All Fields] AND “dust” [MeSH Terms] OR “dust exposure OR “industry” [All Fields] AND “occupational groups” [MeSH Terms] OR “occupational” [All Fields] AND “groups” [All Fields] OR “workers” [All Fields] OR “factories” [All Fields] OR “factory” [All Fields] AND “Africa” [MeSH Terms]. The search aimed to collect published articles without restricting the study period. In this systematic review and meta-analysis, we used advanced searching techniques that combined search terms with Boolean operators such as “AND” and “OR.” The search for all articles was conducted from 10 September 2023 to 20 November 2023.

### Eligibility criteria

#### Inclusion criteria

We formulated research questions and identified eligible primary studies for this systematic review and meta-analysis using the Population, Exposure, Comparator, and Outcome (PECO) criteria. We included studies conducted among workers in organic dust-generating industries in Africa, comparing them to workers in other industries who were not exposed to organic dust. We considered studies that investigated the outcomes of lung function indices such as FVC, FEV1, FEV1/FVC, and PEF in both exposed and unexposed groups. This systematic review and meta-analysis included observational studies (cross-sectional, case–control, and cohort) conducted between 2002 and 2023. This analysis included only full-text articles written in English.

#### Exclusion criteria

We excluded studies with unclear data on the following lung function parameters, such as FVC, FEV1, FEV1/FVC, and PEFR; abstracts without full text; studies discussing inorganic or mixed dust exposure; qualitative research; editorials, commentaries; books; experimental studies; ecological studies; case reports; case series; review articles; and conference proceedings. We also excluded studies that required at least three e-mails to contact the principal investigators of the primary study, as well as articles that could not be obtained in full text.

### Quality assessment and data extraction

Duplicated records were removed from the combined database search results using reference management software (EndNote X8). The data were extracted using a standard data extraction format by two independent reviewers (ZA and HEH). The data extraction spreadsheet format was used, which included the following variables: name of the first author, mean, standard deviation, type of industry, date of publication, country where the study was conducted, and sample size extracted from each study. The titles and abstracts were reviewed, and any records that did not meet the inclusion criteria were excluded from the systematic review and meta-analysis.

The quality of the selected studies was evaluated using the Newcastle–Ottawa Scale (NOS), which has three domains: selection (a maximum of four stars), comparability (a maximum of two stars), and outcome (a maximum of three stars) (45). The quality of all included studies was assessed independently by the authors. When the reviewers disagreed, the issue was discussed, and a third reviewer was invited to resolve any disagreements. In this systematic review and meta-analysis, articles with quality scores ranging from 6 to 9 were included.

### Statistical analysis

The retrieved data were analyzed using STATA 17.0. The *I*^2^ test was used to assess the heterogeneity of the included studies. As there was heterogeneity across the included studies, we conducted a random-effects meta-analysis using the DerSimonian–Laird estimators to estimate the overall mean difference between the exposed and unexposed groups, and the pooled findings were displayed using forest plots. Publication bias was assessed visually using funnel plots and objectively using Egger’s test, with a *p*-value less than 0.05. Subgroup analysis was performed by study country and the type of organic dust to which workers were exposed to reduce the random changes between the primary studies’ inter-group estimates. Moreover, we performed meta-regression to examine correlations between the outcome variable and the selected predictors.

## Results

**Figure 1:**
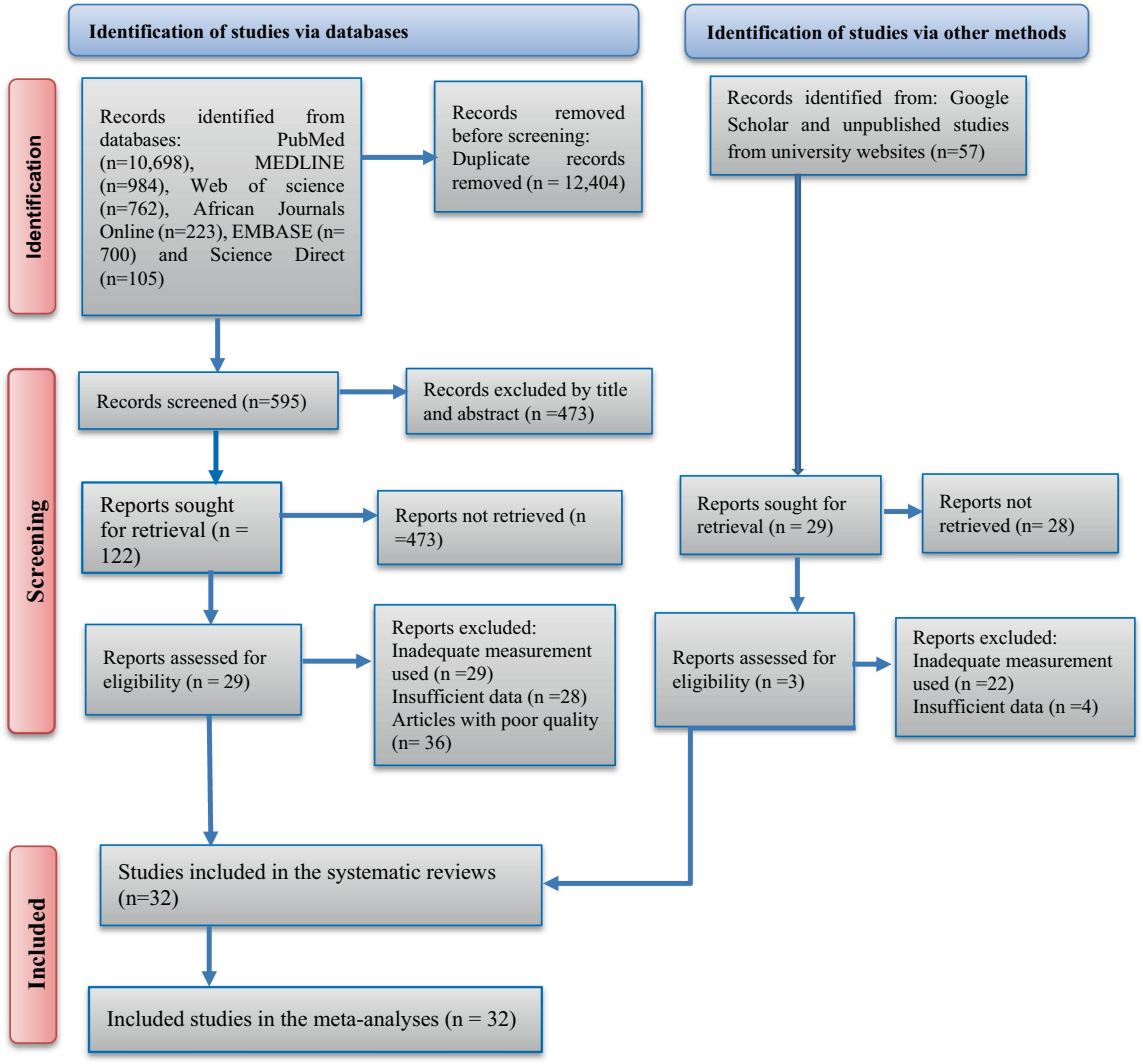
PRISMA flow diagram describes the selection of the studies for the meta-analysis of the lung function parameters among the organic dust-exposed African industrial workers.

We identified a total of 13,529 studies by performing electronic searches in PubMed, MEDLINE, the Web of Science, African Journals Online, Embase, and ScienceDirect, along with 57 studies identified from other sources such as Google Scholar and university websites. Of these, 12,404 duplicate articles were omitted; 473 studies were excluded after reviewing titles/abstracts; and we could not retrieve 473 articles identified through database searches and 28 articles identified from other sources, leading to their removal. Moreover, 29 articles were excluded due to insufficient measurement, 28 articles were not included due to insufficient data, and 36 articles were removed due to poor quality. Furthermore, 22 articles and 4 articles from other sources were excluded due to inadequate measurement and insufficient data, respectively. Finally, this systematic review and meta-analysis included 32 studies that met the eligibility criteria (Figure 1).

### Characteristics of the included studies

This systematic review and meta-analysis included 32 primary studies conducted in Africa and published between 2002 and 2023. The majority of the included studies in this meta-analysis were conducted in Nigeria (11 studies) and Ethiopia (10 studies). The primary studies included a total of 7,085 participants, with 3,914 being organic dust-exposed workers and 3,171 being non-exposed workers.

This systematic review and meta-analysis included primary studies conducted in different work environments and comprised five types of exposure: coffee dust (in 3 studies) (46–48), grain dust (in 3 studies) (42, 49, 50), flour dust (in 8 studies) (25, 28, 29, 36, 51–54), wood dust (in 12 studies) (31, 35, 38–40, 55–61), and cotton dust (in 6 studies) (32, 62–66). The following lung function test parameters were investigated in the included primary studies: FVC in 25 studies, FEV_1_ in 26 studies, FEV_1_/FVC in 24 studies, and PERF in 16 studies (Table 1).

**Table 1:** Characteristics and lung function indices of the 32 primary studies included in the systematic review and meta-analysis to estimate the pooled mean difference among organic dust-exposed industrial workers in Africa.

Author and publication year	Country	Type of organic dust	Study participants		Lung function test parameters								NOQS
			Exposed	Unexposed	FVC (Mean, L)		FEV_1_ (Mean, L)		FEV_1_/FVC (%)		PEFR (mean, L/min)		
					Exposed	Unexposed	Exposed	Unexposed	Exposed	Unexposed	Exposed	Unexposed	
Abaya et al., 2019	Ethiopia	Coffee dust	114	54	3.08	3.33	2.43	2.69	78	81	–	–	8
Abaya et al., 2018	Ethiopia	Coffee dust	104	103	4.03	4.41	3.22	3.63	80	83	–	–	7
Abdulsalam Saliu Tosho, 2015	Egypt	Flour dust	101	101	–	–	–	–	96.67	101.61	516.72	575.37	8
A. J. Ugheoke et al., 2006	Nigeria	Wood dust	86	139	–	–	–	–	–	–	524.93	577.01	7
Nigisti Abraha, 2014	Ethiopia	Wood dust	50	50	3.96	4.65	3.77	4.29	–	–	–	–	6
Adeoye, et al., 2014	Nigeria	Wood dust	50	50	–	–	–	–	–	–	341.80	479.80	6
Asgedom et al., 2019	Ethiopia	Wood dust	74	73	4.96	4.93	4.10	4.12	82.36	83.14	–	–	8
Demeke and Haile, 2018	Ethiopia	Flour dust	54	54	4.25	5.30	3.46	4.50	81.93	83.40	5.43	7.87	8
Derso et al., 2021	Ethiopia	Cotton dust	83	83	2.79	3.13	2.56	3.01	92.20	96.00	4.88	6.25	7
Dunga JA, et al., 2015	Nigeria	Wood dust	200	200	3.45	3.89	2.79	2.84	71.76	82.10	522.93	552.0	9
Ennin et al., 2017	Ghana	Wood dust	104	104	3.46	3.63	2.58	2.90	73.12	79.13	305.43	392.30	9
Fahim and El-Prince, 2013	Egypt	Flour dust	43	64	3.80	4.40	3.40	3.60	87.10	91.20	–	–	6
Fentie et al., 2019	Ethiopia	Wood dust	70	70	3.19	3.70	2.70	3.24	85.08	86.86	5.23	6.01	7
H.A. Mohammadien et al., 2013	Egypt	Flour dust	200	200	–	–	–	–	66.1	76.4 0	–	–	9
Hamed O. Khalifa, 2003	Egypt	Cotton dust	80	90	–	–	–	–	81.67	80.10	–	–	6
Hinson et al., 2014	Benin	Cotton dust	109	107	3.15	3.38	2.45	2.59	–	–	–	–	8
Hinson et al., 2016	Benin	Cotton dust	656	113	3.69	3.64	3.00	3.04	–	–	–	–	9
Ibekwe and Okojie 2014	Nigeria	Flour dust	200	200	2.67	2.76	2.57	2.59	95.75	97.87	409.13	548.93	9
Ige and Awoyemi 2002	Nigeria	Flour dust	100	100	3.69	4.50	3.10	3.50	70.30	78.10	–	–	7
Ijadunola et al., 2005	Nigeria	Flour dust	75	109	4.1	5.40	3.30	4.70	86.00	90.5	478.00	725 0.00	7
Jabur et al., 2022	Ethiopia	Wood dust	50	50	3.82	4.34	3.19	3.65	83.44	84.37	–	–	8
K. Iyogun et al., 2019	Nigeria	Grain Dust	72	72	–	–	1.60	2.10	–	–	186.70	269.50	7
Kanko et al., 2017	Ethiopia	Cotton dust	51	51	2.811	3.70	2.73	3.60	96.63	97.10	5.99	6.90	7
Lagiso et al., 2020	Ethiopia	Flour dust	48	48	2.78	3.19	3.46	3.91	74.10	79.87	–	–	8
Mwelange et al., 2019	Tanzania	Cotton dust	164	161	3.43	4.22	2.50	3.30	82.49	97.84	–	–	9
Obem Okwari, 2005	Nigeria	Wood dust	221	200	3.2	3.90	2.6	3.2	76.53	82.80	385.50	586.70	9
Omigie et al., 2023	Nigeria	Wood dust	180	60	3.82	2.32	2.32	4.01	–	–	1.68	3.25	7
Omole oj et al., 2018	Nigeria	Wood dust	102	102	2.73	3.14	2.47	3.10	–	–	–	–	8
Sakwari et al., 2013	Tanzania	Coffee dust	133	106	3.88	3.84	3.25	3.28	84.10	85.70	–	–	9
Tobin et al., 2015	Nigeria	Wood dust	115	156	3.60	3.79	3.07	3.30	77.64	79.48	404.11	457.40	9
Ulanga et al., 2021	Tanzania	Grain dust	144	20	3.94	3.43	2.80	2.92	74.67	84.07	448.31	468.00	6
Virginia Kimanzi, 2022	Kenya	Grain dust	81	81	–	–	–	–	95.42	113.72	–	–	7

NOQS, Newcastle–Ottawa Quality Score.

The mean difference in FVC between the exposed and unexposed groups was measured in 25 studies, with 18 of them reporting a significant reduction in FVC among the exposed group compared to the non-exposed group. From the included primary studies, the mean difference in FEV_1_ between the exposed and non-exposed groups was evaluated in 26 studies, and all of them reported a reduction in mean FEV_1_ among the exposed group compared to the non-exposed group, with a significant decrease in FEV_1_ in 17 studies.

The mean difference in FEV_1_/FVC was assessed in 24 primary included studies, and the results revealed that 15 of these studies reported statistically significant reductions in the mean difference in FEV_1_/FVC between exposed and non-exposed groups. Furthermore, 14 of the 16 primary studies that evaluated the mean difference in PEFR between the exposed and non-exposed groups showed a statistically significant reduction. We evaluated the quality of the included studies using a modified version of the Newcastle–Ottawa Scale, with the lowest and highest scores being six and nine stars, respectively (Table 1).

**Figure 2:**
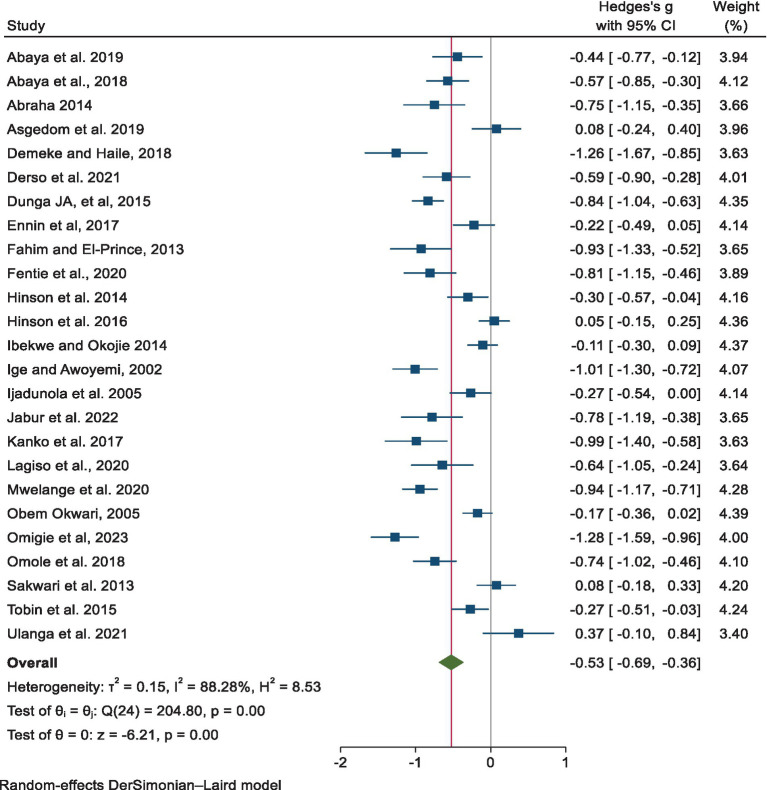
Estimates of the point and pooled mean difference in FVC for exposed and non-exposed industrial workers in Africa.

We observed high heterogeneity among the included primary studies in this systematic review and meta-analysis [*Q* = 204.80, *I*^2^ = 88.82%; *p* < 0.001]. Therefore, we used a random-effects model to estimate the pooled mean difference in FVC between the exposed and non-exposed groups. The pooled estimate of the mean difference in FVC was found to be −0.53 [95% CI: −0.83, −0.36, *p* < 0.001], which was significantly reduced in the exposed group compared to the non-exposed group (Figure 2).

**Figure 3:**
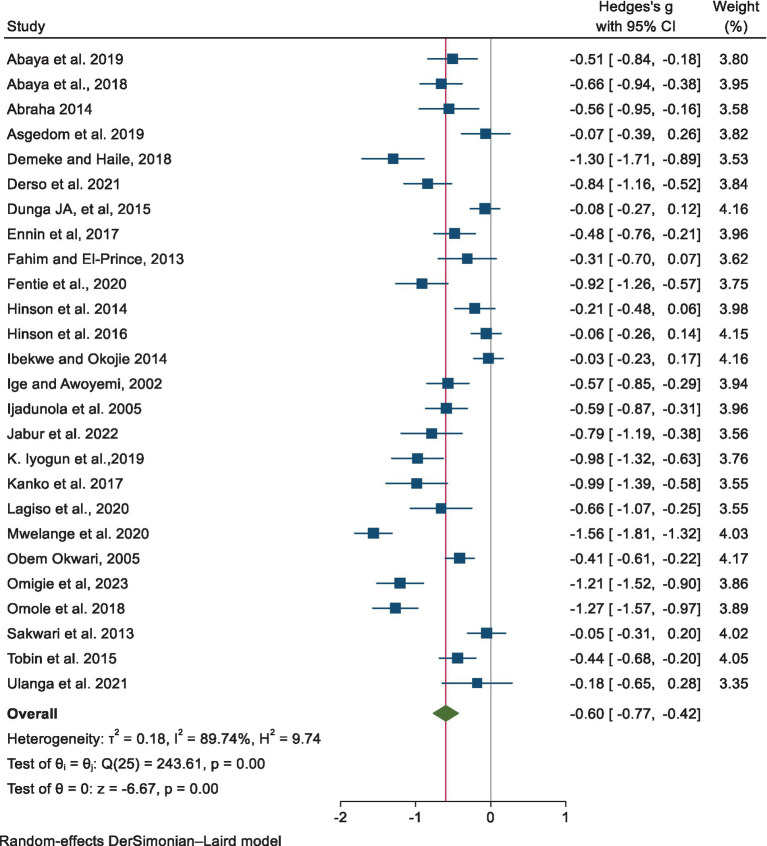
Estimates of the point and pooled mean difference in FEV_1_ among exposed and non-exposed industrial workers in Africa.

The pooled estimate of the mean difference in FEV_1_ using a random-effects model revealed a significant reduction among the exposed group compared to the non-exposed group, with a mean difference of −0.60 [−0.77, −0.42, *p* < 0.001] (Figure 3).

**Figure 4:**
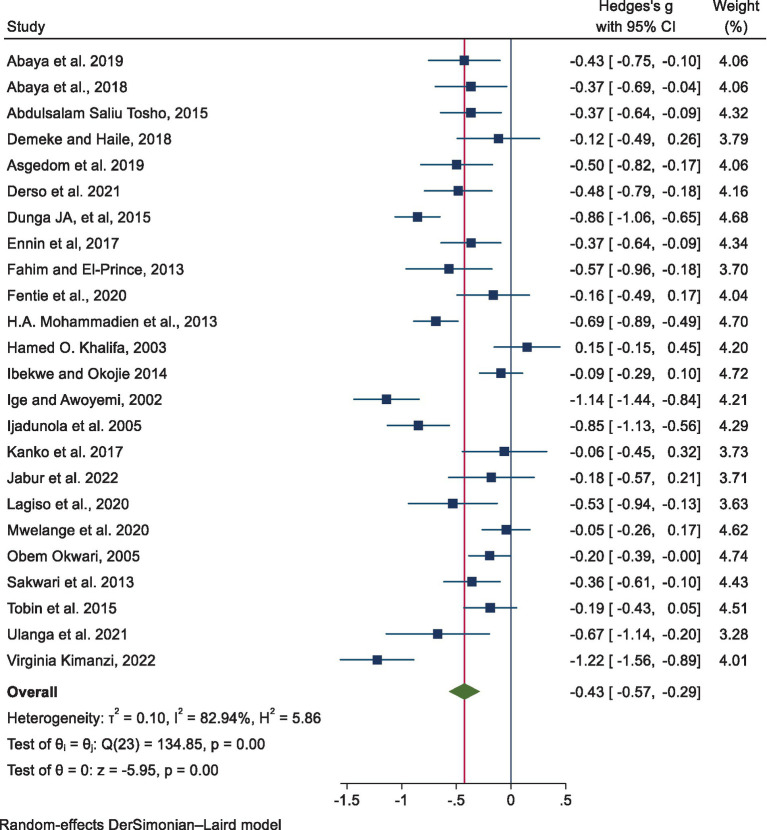
Point and pooled estimates of the mean difference in FEV_1_/FVC among exposed and non-exposed industrial workers in Africa.

The pooled estimates of the mean difference in FEV_1_/FVC using a random-effects model revealed a statistically significant reduction in the exposed group compared to the non-exposed group, with a mean difference of −0.43 [−0.57, −0.29, *p* < 0.001] (Figure 4).

**Figure 5:**
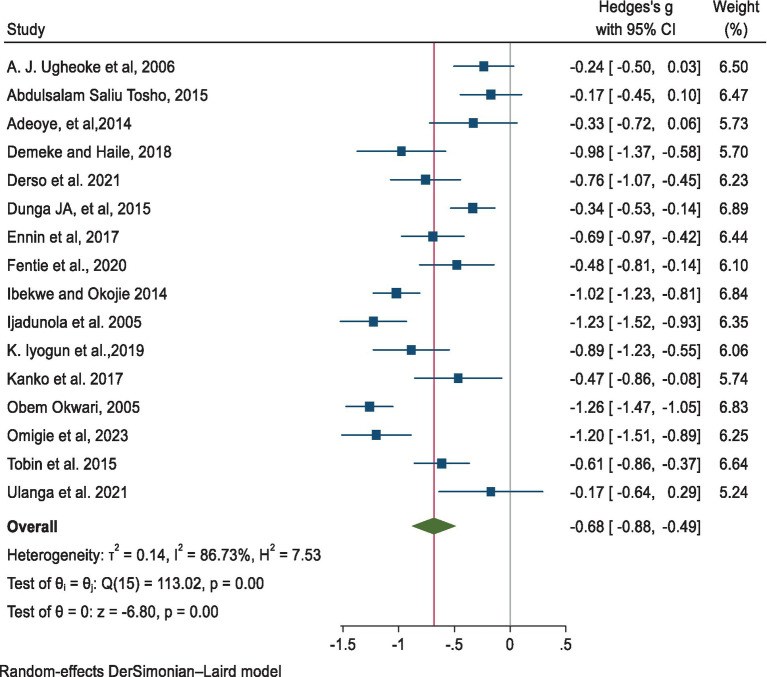
Point and pooled estimates of the mean difference in PEFR between the exposed and non-exposed industrial workers in Africa.

We used a random-effects model in this systematic review and meta-analysis as there was significant heterogeneity between included primary studies [*Q* = 184.08, *I*^2^ = 92.98%; *p* < 0.001]. The result of the analysis revealed that there was a significant reduction in the pooled mean difference in PEFR of −0.69 [95% CI: −0.88, −0.49, *p* < 0.001] among the exposed group compared to the non-exposed group (Figure 5).

### Subgroup analyses

Subgroup analyses were performed based on the type of organic dust to which the workers were exposed and the countries in which the studies were conducted. In a subgroup analysis of 25 studies, workers exposed to flour dust [−0.68, 95% CI: −1.05, −0.32, *p* < 0.000], cotton dust [−0.54, 95% CI: −0.93, −0.15, *p* < 0.006], and wood dust [−0.57, 95% CI: −0.83, −0.31, *p* < 0.000] had significantly lower FVC than non-exposed workers. According to the subgroup analysis by country, there was a statistically significant reduction in FVC among the workers exposed to organic dust compared to those who were not exposed in the studies conducted in Egypt [−0.93, 95% CI: −1.33, −0.52, *p* < 0.000], Ethiopia [−0.66, 95% CI: 0.88, −0.44, *p* < 0.000], and Nigeria [−0.58, 95% CI: −0.88, −0.28, *p* < 0.000].

A subgroup analysis of the lung function parameter of FEV1 by type of organic dust showed that the workers exposed to dust had significantly lower FEV1 than non-exposed workers for coffee dust [−0.40, 95% CI:-0.77, −0.03, *p* < 0.032], cotton dust [−0.73, 95% CI: −1.27, −0.18, *p* < 0.009], flour dust [−0.56, 95% CI: −0.89, −0.22, *p* < 0.001], and wood dust [−0.61, 95% CI: −0.88, −0.35, *p* < 0.000]. In addition, a subgroup analysis of FEV1 by country revealed that in the studies conducted in Ethiopia, Nigeria, and Ghana, the mean difference in FEV1 was statistically significantly lower among organic dust-exposed workers than among non-exposed workers.

A subgroup analysis of 24 included studies revealed that, except for cotton dust, FEV1/FVC was statistically significantly reduced among the workers exposed to different types of dust, with grain dust showing the highest mean difference. In every country where the studies were conducted, except for Tanzania, the FEV_1_/FVC ratio was statistically significantly lower among workers exposed to dust compared to non-exposed workers. A subgroup analysis of the lung function parameter PEFR by type of organic dust revealed a statistically significant reduction in the workers exposed to wood dust [−0.65, 95% CI: −0.93, −0.37, *p* < 0.000], cotton dust [−0.64, 95% CI: −0.92, −0.35, *p* < 0.000], and flour dust [−0.85, 95% CI: −1.31, −0.39, *p* < 0.000] compared to the non-exposed workers. Furthermore, a subgroup analysis by country revealed that the studies conducted in Ethiopia, Ghana, and Nigeria showed a statistically significant reduction in PEFR among organic dust-exposed workers compared to non-exposed workers (Table 2).

**Table 2:** Subgroup analysis of the lung function indices—FVC, FEV_1_, FEV_1_/FVC, and PEFR—among organic dust-exposed industrial workers in Africa.

Variables	Category	Included studies	Pooled mean difference	*p*-value
**FVC**				
Type of organic dust	Coffee dust	3	−0.31 [−0.71, 0.09]	0.131
	Cotton dust	5	−0.54 [−0.93, −0.15]	0.006*
	Flour dust	6	−0.68 [−1.05, −0.32]	0.000*
	Grain dust	1	0.37 [−0.10, 0.84]	0.120
	Wood dust	10	−0.57 [−0.83, −0.31]	0.000*
**FEV**_**1**_				
Type of organic dust	Coffee dust	3	−0.40 [−0.77, −0.03]	0.032*
	Cotton dust	5	−0.73 [−1.27, −0.18]	0.009*
	Flour dust	6	−0.56 [−0.89, −0.22]	0.001*
	Grain dust	2	−0.59 [−1.37, 0.18]	0.134
	Wood dust	10	−0.61 [−0.88, −0.35]	0.000*
**FEV**_**1**_ **/FVC**				
Type of organic dust	Coffee dust	3	−0.38 [−0.55, −0.21]	0.000*
	Cotton dust	4	−0.11 [−0.37, 0.15]	0.411
	Flour dust	8	−0.54 [−0.80, −0.29]	0.000*
	Grain dust	2	−0.97 [−1.51, −0.43]	0.000*
	Wood dust	7	−0.36 [−0.56, −0.16]	0.001*
**PEFR**				
Type of organic dust	Cotton dust	2	−0.64 [−0.92, −0.35]	0.000*
	Flour dust	4	−0.85 [−1.31, −0.39]	0.000*
	Grain dust	2	−0.55 [−1.25, 0.15]	0.124
	Wood dust	8	−0.65 [−0.93, −0.37]	0.000*
**FVC**				
Study country	Benin	2	−0.12 [−0.46, 0.23]	0.508
	Egypt	1	−0.93 [−1.33, −0.52]	0.000*
	Ethiopia	10	−0.66 [−0.88, −0.44]	0.000*
	Ghana	1	−0.22 [−0.49, 0.05]	0.109
	Nigeria	8	−0.58 [−0.88, −0.28]	0.000*
	Tanzania	3	−0.18 [−0.96, 0.60]	0.650
**FEV**_**1**_				
Study country	Benin	2	−0.11 [−0.27, 0.05]	0.163
	Egypt	1	−0.31 [−0.70, 0.07]	0.114
	Ethiopia	10	−0.72 [−0.92, −0.51]	0.000*
	Ghana	1	−0.48 [−0.76, −0.21]	0.001*
	Nigeria	9	−0.61 [−0.90, −0.31]	0.000*
	Tanzania	3	−0.61 [−1.57, 0.35]	0.214
**FEV**_**1**_ **/FVC**				
Study country	Egypt	4	−0.37 [−0.74, −0.01]	0.046*
	Ethiopia	9	−0.33 [−0.44, −0.21]	0.000*
	Kenya	1	−1.22 [−1.56, −0.89]	0.000*
	Nigeria	6	−0.55 [−0.90, −0.19]	0.003*
	Tanzania	3	−0.31 [−0.64, 0.02]	0.065
	Ghana	1	−0.37 [−0.64, 0.09]	0.009*
**PEFR**				
Study country	Egypt	1	−0.17 [−0.45, 0.10]	0.220
	Ethiopia	4	−0.67 [−0.89, −044]	0.000*
	Ghana	1	−0.69 [−0.97, −0.42]	0.000*
	Nigeria	9	−0.79 [−1.07, −0.52]	0.000*
	Tanzania	1	−0.17 [−0.64, 0.29]	0.465

*Statistically significant variation.

### Publication bias

**Figure 6:**
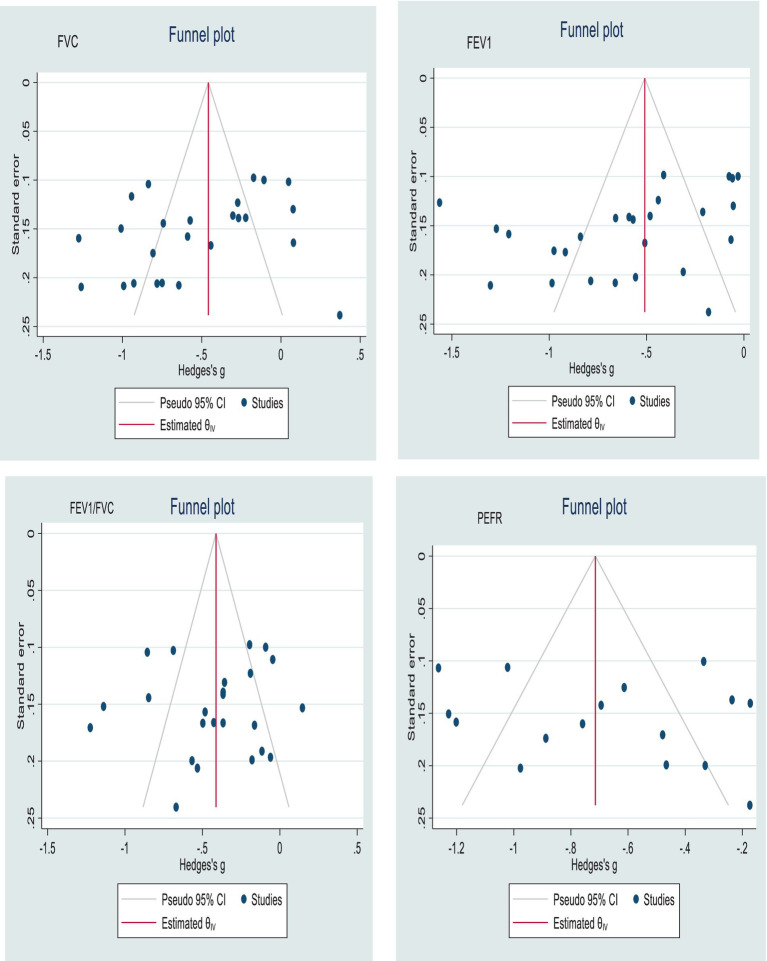
Funnel plots with 95% confidence intervals show the pooled mean difference in FVC, FEV_1_, FEV_1_/FVC, and PEFR among industrial workers in Africa.

The publication bias was assessed subjectively using funnel plots, and the findings revealed a slightly asymmetrical distribution of studies, indicating the presence of publication bias (Figure 6). However, the findings of Egger’s test showed that there was no statistically significant publication bias for all lung function tests (Table 3).

**Table 3:** Results of publication bias in lung function tests among African industrial workers.

Lung function test	Number of studies	Publication bias (Egger’s test)	
		β-coefficient	*p*-value
FVC	25	−3.19	0.135
FEV_1_	26	−3.76	0.078
FEV_1_/FVC	24	−0.70	0.724
PEFR	16	2.49	0.333

### Meta-regression

Meta-regression was used to determine the association between lung function in African industrial workers and several variables, such as the type of organic dust they were exposed, the country where the study was conducted, and the year in which it was published. The findings revealed that there was no statistically significant association between the outcome variables and predictors (Table 4).

**Table 4:** Results of meta-regression of lung function tests among African industrial workers.

Variables	Lung function indices	β-coefficient	*p*-value	[95% conf. interval]
Study country	FVC	0.084	0.070	[−0.007–0.175]
	FEV1	0.044	0.345	[−0.048–0.136]
	FEV1/FVC	−0.015	0.792	[−0.129–0.099]
	PEFR	0.094	0.212	[−0.054–0.242]
Year of publication	FVC	−0.012	0.480	[−0.045–0.021]
	FEV1	−0.029	0.060	[−0.059–0.001]
	FEV1/FVC	0.004	0.742	[−0.021–0.030]
	PEFR	0.011	0.523	[−0.023–0.046]
Type of organic dust	FVC	0.052	0.214	[−0.061–0.173]
	FEV1	0.012	0.839	[−0.104–0.128]
	FEV1/FVC	−0.037	0.526	[−0.157–0.082]
	PEFR	0.011	0.910	[−0.176–0.198]

## Discussion

Organic dust exposure in the workplace can be hazardous to the respiratory health of industrial workers. Inhaling organic dust, such as cotton dust, wood dust, flour dust, paper dust, grain dust, animal confinement dust, or compost dust, can lead to inflammatory responses in the respiratory system and airway obstruction among workers in dusty environments (15, 16). The objective of this systematic review and meta-analysis was to determine the pooled mean difference in lung function indices among African industrial workers exposed to organic dust. Accordingly, the pooled mean differences in FVC, FEV_1_, FEV_1_/FVC ratio, and PEFR between organic dust-exposed industrial workers and non-exposed industrial workers were found to be 0.53 L, 0.60 L, 0.47, and 0.69 L/min lower, respectively.

In this systematic review and meta-analysis, the pooled mean difference in FVC, FEV_1_, FEV_1_/FVC ratio, and PEFR was statistically significantly reduced among the organic dust-exposed workers compared to non-exposed workers. According to the findings, FVC statistically significantly reduced among organic dust-exposed workers compared to non-exposed workers. The findings of this systematic review and meta-analysis is consistent with the outcomes of studies on coffee dust-exposed workers in Ethiopia and Tanzania (67), as well as in India (68–70), Pakistan (71), and Iran (72). Furthermore, the current findings are consistent with a systematic review and meta-analysis conducted in Ethiopia among workers exposed to organic and inorganic dust in the workplace (73). However, the findings of this study were inconsistent with another systematic review and meta-analysis conducted in industrialized countries, which found no statistically significant relationship between organic dust exposure and FVC (19), as well as studies conducted in Macedonia (74) and India (75). The variations could be attributed to differences in study design, duration of exposure, personal protective use, sample size, and workplace dust concentration.

The pooled mean estimate of FEV_1_ was statistically significantly lower among organic dust-exposed workers compared to non-exposed workers. This finding is consistent with the findings of a systematic review and meta-analysis conducted in industrialized countries (19), as well as research conducted in Ethiopia and Tanzania (67), India (68–70), Pakistan (71), and Iran (76). Furthermore, it is also consistent with a systematic review and meta-analysis conducted in Ethiopia among workers exposed to both organic and inorganic dust (73). However, a study conducted in Iran contradicted this systematic review and meta-analysis (72).

A study conducted on coffee dust-exposed workers revealed that the ratio of FEV_1_/FVC was not statistically significantly lower among dust-exposed workers compared to non-exposed workers (67). However, it was statistically significantly lower among organic dust-exposed workers compared to non-exposed workers in this study, which is consistent with studies conducted in India (70), Iran (76), Macedonia (74), and Greece (77). The disparity could be explained by differences in sample size, duration of exposure, dust levels in working environments, a lack of dust control measures, inadequate ventilation, smoking status of exposed workers, and a lack of work environment modification measures.

Furthermore, the current systematic review and meta-analysis found that PEFR in organic dust-exposed workers was statistically significantly lower than in the non-exposed workers. This finding is consistent with a systematic review and meta-analysis of Ethiopian workers exposed to both organic and inorganic dust at work (73). Furthermore, the current study’s findings agree with the results of previous studies conducted in India (68–70, 72, 75), Iran (72), and Pakistan (78). Possible explanations for the reduction in the lung function parameters among the organic dust-exposed industrial workers include the exposure to various dust particles in the workplace due to a lack and improper use of personal protective equipment, a lack of dust control measures, and a lack of improved ventilation, which leads to increased dust accumulation in the respiratory system due to prolonged exposure to organic dust in work environments.

The subgroup analysis revealed that exposure to organic dust, including cotton, coffee, grain, flour, and wood dusts, produced inconsistent results in terms of the association between exposure to organic dust and changes in lung function indices. In subgroup analysis, exposure to wood and flour dust was most consistently associated with all lung function indices; however, not all exposure to wood and flour dust in primary studies showed a statistically significant association. Furthermore, another meta-analysis revealed that exposure to grain dust was the most consistently associated with all lung function indices (4). However, it is difficult to determine whether any particular type of exposure was more evidently associated with a reduction in lung function. Differences in sample size, duration of exposure, work environment, study design, and study settings could all contribute to the observed difference.

### Limitations of the study

The following limitations should be considered when interpreting the findings of this systematic review and meta-analysis. The first limitation is that the included studies vary significantly, which could be due to differences in exposure level, duration of exposure, use of personal protective equipment, and neglect of confounders and demographic differences in the primary studies. To address this heterogeneity, we used a random-effects model. The second limitation is that the studies included in this systematic review and meta-analysis were cross-sectional, limiting the ability to draw causal inferences about the long-term effects of organic dust exposure on lung function.

The third limitation is that the included studies were only published in English; similar studies conducted in other languages were excluded. The fourth limitation is that the studies included in this systematic review and meta-analysis were conducted in only six African countries; therefore, they did not sufficiently represent the rest of Africa. Finally, some studies had small sample sizes, which may have an effect on the pooled estimate of lung function indices.

## Conclusion

The findings of this systematic review and meta-analysis revealed that exposure to organic dust contributes to the reduction in lung function parameters—FVC, FEV1, FEV1/FVC, and PEFR—among workers in African industries that produce organic dust.

## Recommendations

- Implementing dust control measures and evaluating the effectiveness of different dust control interventions, such as improved ventilation, personal protective equipment use, and work environment modifications, are critical for reducing dust exposure and improving respiratory health outcomes.
- Further research is necessary to determine which types of organic dust are more harmful or have specific health effects in order to prioritize interventions and regulatory measures.
- Sensitive techniques, such as impulse oscillometry and high-resolution computed tomography (HRCT), can be employed to detect early small airway dysfunction and provide a comprehensive picture of the respiratory impact of dust exposure.
- Furthermore, longitudinal studies could evaluate long-term health outcomes for workers exposed to different types of organic dust.

## References

[1] Kanageswari SV Tabil LG Sokhansanj S. Dust and particulate matter generated during handling and Pelletization of herbaceous biomass: A review. Energies. (2022) 15:2634. doi: 10.3390/en15072634

[2] Rushton L. Organic dusts and respiratory cancer: a complex issue. Occup Environ Med. (2006) 63:717. doi: 10.1136/oem.2006.028316, PMID: 17050744 PMC2077987

[3] Susan P Hans K Ann CO Heinz-Erich W Irene B Dario C . Occupational exposure to organic dust increases lung cancer risk in the general population. Thorax. (2012) 67:111. doi: 10.1136/thoraxjnl-2011-200716 21856697 PMC8363447

[4] Anneli Clea B Martin RM Torben S Vivi S. The effect of organic dust exposure on change in lung function – A systematic review. Eur Respir J. (2016) 48:PA1169. doi: 10.1183/13993003.congress-2016.PA1169

[5] Shivpuje S Mehta A Patil D Dharaiya P. Evaluation of organic and inorganic dust concentration in different mechanized agricultural operations for wheat crop. Int J Curr Microbiol App Sci. (2020) 9:2806–13. doi: 10.20546/ijcmas.2020.907.331

[6] Omland Ø. Exposure and respiratory health in farming in temperate zones--a review of the literature. Ann Agric Environ Med. (2002) 9:119–36. PMID: 12498578

[7] Kim KH Kabir E Kabir S. A review on the human health impact of airborne particulate matter. Environ Int. (2015) 74:136–43. doi: 10.1016/j.envint.2014.10.005 25454230

[8] Terzano C Di Stefano F Conti V Graziani E Petroianni A. Air pollution ultrafine particles: toxicity beyond the lung. Eur Rev Med Pharmacol Sci. (2010) 14:809–21. PMID: 21222367

[9] Tran VV Park D. Indoor air pollution, related human diseases, and recent trends in the control and improvement of indoor air quality. Int J Environ Res Public Health. (2020) 17:2927. doi: 10.3390/ijerph17082927 32340311 PMC7215772

[10] Simpson JC Niven RM Pickering CA Fletcher AM Oldham LA Francis HM. Prevalence and predictors of work related respiratory symptoms in workers exposed to organic dusts. Occup Environ Med. (1998) 55:668–72. doi: 10.1136/oem.55.10.668, PMID: 9930087 PMC1757512

[11] Lacey J Dutkiewicz J. Bioaerosols and occupational lung disease. J Aerosol Sci. (1994) 25:1371–404. doi: 10.1016/0021-8502(94)90215-1

[12] Matheson MC Benke G Raven J Sim MR Kromhout H Vermeulen R . Biological dust exposure in the workplace is a risk factor for chronic obstructive pulmonary disease. Thorax. (2005) 60:645–51. doi: 10.1136/thx.2004.035170, PMID: 16061705 PMC1747486

[13] Douwes J Thorne P Pearce N Heederik D. Bioaerosol health effects and exposure assessment: progress and prospects. Ann Occup Hyg. (2003) 47:187–200. doi: 10.1093/annhyg/meg032, PMID: 12639832

[14] Vested A Basinas I Burdorf A Elholm G Heederik DJJ Jacobsen GH . A nationwide follow-up study of occupational organic dust exposure and risk of chronic obstructive pulmonary disease (COPD). Occup Environ Med. (2019) 76:105–13. doi: 10.1136/oemed-2018-105323, PMID: 30598459 PMC6581073

[15] Rylander R. Organic dusts and lung reactions--exposure characteristics and mechanisms for disease. Scand J Work Environ Health. (1985) 11 Spec No):199-206:199–206. doi: 10.5271/sjweh.2234 4035322

[16] Castranova V Robinson VA Frazer DG. Pulmonary reactions to organic dust exposures: development of an animal model. Environ Health Perspect. (1996) 104:41–53. doi: 10.1289/ehp.96104s141 8722109 PMC1469574

[17] Malmberg P. Health effects of organic dust exposure in dairy farmers. Am J Ind Med. (1990) 17:7–15. doi: 10.1002/ajim.4700170104, PMID: 2407117

[18] Poole JA Romberger DJ. Immunological and inflammatory responses to organic dust in agriculture. Curr Opin Allergy Clin Immunol. (2012) 12:126–32. doi: 10.1097/ACI.0b013e3283511d0e, PMID: 22306554 PMC3292674

[19] Bolund AC Miller MR Sigsgaard T Schlünssen V. The effect of organic dust exposure on long-term change in lung function: a systematic review and meta-analysis. Occup Environ Med. (2017) 74:531–42. doi: 10.1136/oemed-2016-103963, PMID: 28404791

[20] Post W Heederik D Houba R. Decline in lung function related to exposure and selection processes among workers in the grain processing and animal feed industry. Occup Environ Med. (1998) 55:349–55. doi: 10.1136/oem.55.5.349 9764113 PMC1757584

[21] Eduard W Pearce N Douwes J. Chronic bronchitis, COPD, and lung function in farmers: the role of biological agents. Chest. (2009) 136:716–25. doi: 10.1378/chest.08-2192 19318669

[22] Bolund AC Miller MR Basinas I Elholm G Omland Ø Sigsgaard T . The effect of occupational farming on lung function development in young adults: a 15-year follow-up study. Occup Environ Med. (2015) 72:707–13. doi: 10.1136/oemed-2014-102726, PMID: 26265668

[23] Tefera Zele Y Kumie A Deressa W Moen BE Bråtveit M. Reduced cross-shift lung function and respiratory symptoms among integrated textile factory Workers in Ethiopia. Int J Environ Res Public Health. (2020) 17:2741. doi: 10.3390/ijerph17082741, PMID: 32316175 PMC7215879

[24] Oo TW Thandar M Htun YM Soe PP Lwin TZ Tun KM . Assessment of respiratory dust exposure and lung functions among workers in textile mill (Thamine), Myanmar: a cross-sectional study. BMC Public Health. (2021) 21:673. doi: 10.1186/s12889-021-10712-0, PMID: 33827504 PMC8028193

[25] Demeke D Haile DW. Assessment of respiratory symptoms and pulmonary function status among Workers of Flour Mills in Addis Ababa, Ethiopia: comparative cross-sectional study. Pulmon Med. (2018) 2018:1–7. doi: 10.1155/2018/9521297 PMC6220375 30473887

[26] Hamdy Ali Mohammadien M Mona Taha H. Raaft Talaat I: effects of exposure to flour dust on respiratory symptoms and pulmonary function of mill workers. Eur Respir J. (2012) 40:P1032.

[27] Douwes J Cheng S Travier N Cohet C Niesink A McKenzie J . Farm exposure in utero may protect against asthma, hay fever and eczema. Eur Respir J. (2008) 32:603–11. doi: 10.1183/09031936.00033707, PMID: 18448493

[28] Lagiso ZA Mekonnen WT Abaya SW Takele AK Workneh HM. Chronic respiratory symptoms, lung function and associated factors among flour mill factory workers in Hawassa city, southern Ethiopia: “comparative cross-sectional study”. BMC Public Health. (2020) 20:909. doi: 10.1186/s12889-020-08950-9, PMID: 32527249 PMC7291423

[29] Mohammadien HA Hussein MT El-Sokkary RT. Effects of exposure to flour dust on respiratory symptoms and pulmonary function of mill workers. Egypt J Chest Dis Tuberculosis. (2013) 62:745–53. doi: 10.1016/j.ejcdt.2013.09.007

[30] Andersson E Sällsten G Lohman S Neitzel R Torén K. Lung function and paper dust exposure among workers in a soft tissue paper mill. Int Arch Arbeitsmed. (2020) 93:105–10. doi: 10.1007/s00420-019-01469-6, PMID: 31451924 PMC6989582

[31] Jabur B Ashuro Z Abaya SW. Chronic respiratory symptoms and lung function parameters in large-scale wood factory workers in Addis Ababa, Ethiopia: a comparative cross-sectional study. Int Arch Occup Environ Health. (2022) 95:1221–30. doi: 10.1007/s00420-022-01857-5, PMID: 35362758

[32] Derso Y Dagnew B Akalu Y Getu AA Getnet M Yeshaw Y. Pulmonary function, respiratory symptoms and associated factors among cotton-ginning workers at Gondar city, Northwest Ethiopia: a comparative cross-sectional study. Int J Physiol Pathophysiol Pharmacol. (2021) 13:140–147. PMID: 34868464 PMC8611241

[33] Tageldin MA Gomaa AA Hegazy EAM. Respiratory symptoms and pulmonary function among cotton textile workers at Misr company for spinning and weaving EL-Mahalla, Egypt. Egypt J Chest Dis Tuberculosis. (2017) 66:369–76. doi: 10.1016/j.ejcdt.2017.03.004

[34] Said AM AbdelFattah EB. Almawardi A-AM: effects on respiratory system due to exposure to wheat flour. Egypt J Chest Dis Tuberculosis. (2017) 66:537–48. doi: 10.1016/j.ejcdt.2016.11.006

[35] Ugheoke AJ Ebomoyi MI Iyawe VI. Influence of smoking on respiratory symptoms and lung function indices in sawmill workers in Benin City, Nigeria. Nigerian J Physiol Sci. (2006) 21:49–54. doi: 10.4314/njps.v21i1-2.53957, PMID: 17242718

[36] Ijadunola KT Erhabor GE Onayade AA Ijadunola MY Fatusi AO Asuzu MC. Pulmonary functions of wheat flour mill workers and controls in Ibadan, Nigeria. Am J Ind Med. (2005) 48:308–17. doi: 10.1002/ajim.20219 16167348

[37] Bosan IB Okpai JU. Respiratory sysmptom and ventilatory function among woodworkers in the Savanah Belt of Nigeria. Anal of Africa Medicine. (2004) 3:22–27.

[38] Ennin IE Adzaku FK Dodoo D Adukpo S Antwi-Boasiako C Antwi DA. A study of lung function indices of woodworkers at the Accra timber market in Ghana. Donnish Journal of Medicine and Medical Sciences. (2015) 2:120–124.

[39] Tobin E Ediagbonya T. Occupational exposure to wood dust and respiratory health status of sawmill Workers in South-South Nigeria. J Pollut Effects Control. (2015) 4:04. doi: 10.4172/2375-4397.1000154

[40] Omole J Fabunmi A Akosile C. Respiratory function of sawmill workers and their relationship to duration of exposure to WOOD dust seen in Nigeria. J Environ Occupat Sci. (2018) 7:9–16. doi: 10.5455/jeos.20180403065108

[41] Osuchukwu NC Osuchukwu EC Eko JE Otareh OO. Occupational exposure to wood dust in calabar municipality, cross river state, Nigeria. Int J Sci Res. (2015) 4:1414–20.

[42] Iyogun K Lateef SA Ana G. Lung function of grain millers exposed to grain dust and diesel exhaust in two food Markets in Ibadan Metropolis, Nigeria. Safety Health Work. (2019) 10:47–53. doi: 10.1016/j.shaw.2018.01.002, PMID: 30949380 PMC6428965

[43] Liberati A Altman DG Tetzlaff J Mulrow C Gøtzsche PC Ioannidis JP . The PRISMA statement for reporting systematic reviews and meta-analyses of studies that evaluate health care interventions: explanation and elaboration. J Clin Epidemiol. (2009) 62:e1–e34. doi: 10.1016/j.jclinepi.2009.06.006, PMID: 19631507

[44] Tufanaru C Munn Z Aromataris E Campbell J Hopp L. Chapter 3: systematic reviews of effectiveness. In: E Aromataris Z Munn, editors. JBI manual for evidence synthesis. JBI (2020).

[45] Wells GA Shea B O’Connell Da Peterson J Welch V Losos M . The Newcastle-Ottawa scale (NOS) for assessing the quality of nonrandomised studies in Meta-analyses. Oxford: The Ottawa Hospital Research Institute (2014).

[46] Abaya SW Bråtveit M Deressa W Kumie A Moen BE. Respiratory health among hand pickers in primary coffee-processing factories of Ethiopia. J Occup Environ Med. (2019) 61:565–71. doi: 10.1097/JOM.0000000000001613 31045853

[47] Abaya SW Bråtveit M Deressa W Kumie A Moen BE. Reduced lung function among Workers in Primary Coffee Processing Factories in Ethiopia: A cross sectional study. Environ Res Public Health. (2018) 15:11. doi: 10.3390/ijerph15112415, PMID: 30384429 PMC6266034

[48] Sakwari G Mamuya S Bråtveit M Moen B. Respiratory symptoms, exhaled nitric oxide, and lung function among Workers in Tanzanian Coffee Factories. J Occupat Environ Med. (2013) 55:544–51. doi: 10.1097/JOM.0b013e318285f453 23618889

[49] Kimanzi V Mburu C Njogu P. Effect of exposure to grain dust on pulmonary function of selected animal feed mill Workers in Kiambu County, Kenya In: Sustainable research and innovation conference. JKUAT Main Campus, Kenya: (2022)

[50] Ulanga AJ Mamuya SH Sakwari G Mlimbila J. Respiratory symptoms, lung function and dust exposure among workers in grain milling industries in Dar Es Salaam, Tanzania. MOJ Public Health. (2021) 10:23–9. doi: 10.15406/mojph.2021.10.00354

[51] Tosho AS Adeshina AI Salawu M Tope AJ. Prevalence of respiratory symptoms and lung function of flour mill workers in Ilorin, North Central Nigeria. Int. J. Res. Rev. (2015) 2:355–364.

[52] Fahim AE El-Prince M. Pulmonary function impairment and airway allergy among workers in traditional bakeries. Int J Occup Med Environ Health. (2013) 26:214–9. doi: 10.2478/s13382-013-0082-6, PMID: 23559139

[53] Ibekwe RU Okojie OH. Lung function indices of flour millworkers in Edo and Delta states. Niger Postgrad Med J. (2014) 21:5–10. doi: 10.4103/1117-1936.163662, PMID: 24887244

[54] Ige OM Awoyemi OB. Respiratory symptoms and ventilatory function of the bakery workers in Ibadan, Nigeria. West Afr J Med. (2002) 21:316–8. doi: 10.4314/wajm.v21i4.28009, PMID: 12665275

[55] Abraha N. Pulmonary function test and pulseoximetry in chipwood factry workers of Maichew, Tigray Region. (2014). Available at: http://etd.aau.edu.et/handle/123456789/6401.

[56] Asgedom AA Bråtveit M Moen BE. High prevalence of respiratory symptoms among particleboard Workers in Ethiopia: A cross-sectional study. Int J Environ Res Public Health. (2019) 16:2158. doi: 10.3390/ijerph16122158, PMID: 31216746 PMC6617153

[57] Adeoye O Adeomi A Olugbenga-Bello A Bamidele J Abodunrin O Sabageh O. Respiratory symptoms and peak expiratory flow among sawmill workers in south western Nigeria. J Environ Occupat Sci. (2014) 3:141. doi: 10.5455/jeos.20140205093223

[58] Dunga J Alkali N Mohammed A Adamu Y Bakki B Kida I. Chronic obstructive pulmonary disease as measured by FEV1, FVC, and FEV1/FVC ratio among saw mill workers in Jos, northern Nigeria. Nigerian Health J. (2015) 15:1–8. doi: 10.60787/tnhj.v15i1.182

[59] Okwari OO Antai AB Owu DU Peters EJ Osim EE. Lung function status of workers exposed to wood dust in timber markets in Calabar, Nigeria. Afr J Med Med Sci. (2005) 34:141–5. PMID: 16749338

[60] Omigie MI Iyawe VI Agoreyo FO. Effect of environmental dust pollution on lung function of adult male exposed to sawdust. Am. J. Res. Commun. (2023) 11:22–41.

[61] Fentie D Gebretsadik T Gessesse E Bayula D Dama B Adi W. Effect of occupational wood dust on pulmonary function among woodworkers in Jimma town, Southwest Ethiopia, A comparative cross sectional study. EC Pulmonol Respirat Med. (2019) 8:587–93.

[62] Hinson AV Lokossou VK Schlünssen V Agodokpessi G Sigsgaard T Fayomi B. Cotton dust exposure and respiratory disorders among textile Workers at a Textile Company in the southern part of Benin. Int J Environ Res Public Health. (2016) 13:895. doi: 10.3390/ijerph13090895, PMID: 27618081 PMC5036728

[63] Hinson AV Schlünssen V Agodokpessi G Sigsgaards T Fayomi B. The prevalence of byssinosis among cotton workers in the north of Benin. Int J Occup Environ Med. (2014) 5:194–200. PMID: 25270009 PMC7767605

[64] Mwelange L Mamuya S Sakwari G. Dust exposure and byssinosis among cotton textile workers in Dar ES salaam, Tanzania. MOJ Public Health. (2020) 9:217–21. doi: 10.15406/mojph.2020.09.00349

[65] Tesfaye K Getahun S Gebremeskel F Boda B Eyayu G. Assessment of respiratory status among workers exposed to cotton dust at Arba Minch textile factory, southern Ethiopia, 2017. Int J Med Med Sci. (2017) 9:126–36. doi: 10.5897/IJMMS2017.1327

[66] Khalifa HO Darwish K El-Hady AA El-Baset EA EL-Deen RS Mohamadein H. Pulmonary function tests and respiratory symptoms among cotton ginning workers in southern Egypt (SOHAG). AAMJ. (2003) 1.

[67] Bråtveit M Abaya SW Sakwari G Moen BE. Dust exposure and respiratory health among Workers in Primary Coffee Processing Factories in Tanzania and Ethiopia. Front Public Health. (2021) 9:730201. doi: 10.3389/fpubh.2021.730201, PMID: 34616708 PMC8488214

[68] Dhanasree Naidu V Sankalp Naidu V Sudheer Dwarak P Supriya Sree P Deepthi N. Effect of cotton dust on pulmonary function among cotton textile workers. MRIMS J Health Sci. (2014) 2:72–2. doi: 10.4103/2321-7006.302691

[69] Wagh N Pachpande B Attarde S Ingle S. The influence of workplace environment on lung function of flour mill Workers in Jalgaon Urban Center. J Occup Health. (2006) 48:396–401. doi: 10.1539/joh.48.396, PMID: 17053307

[70] Ekambaram G Vara A Nileshkumar SM Sivasubramanian N. Effect of cotton dust on lungs among female workers in cotton industry in northern Gujarat, India. Bioinformation. (2022) 18:255–60. doi: 10.6026/97320630018255, PMID: 36518136 PMC9722434

[71] Meo SA. Lung function in Pakistani wood workers. Int J Environ Health Res. (2006) 16:193–203. doi: 10.1080/09603120600641375, PMID: 16611564

[72] Bagheri Hosseinabadi M Sc H Krozhdeh J Sc M Khanjani N Zamani A . Relationship between lung function and flour dust in flour factory workers original article. J Commun Health Res. (2013) 2:138–46.

[73] Demeke D Tesfa E. Prevalence of obstructive lung patterns and actual spirometric result at different workplaces in Ethiopia: A systematic review and meta-analysis. Health Sci Rep. (2023) 6:e1359. doi: 10.1002/hsr2.1359, PMID: 37359412 PMC10288973

[74] Bislimovska D Petrovska S Minov J. Respiratory symptoms and lung function in never-smoking male workers exposed to hardwood dust. Open Access Maced J Med Sci. (2015) 3:500–5. doi: 10.3889/oamjms.2015.086, PMID: 27275278 PMC4877847

[75] Dangi BM Bhise AR. Cotton dust exposure: analysis of pulmonary function and respiratory symptoms. Lung India. (2017) 34:144–9. doi: 10.4103/0970-2113.201319, PMID: 28360462 PMC5351356

[76] K Hosseini D Malekshahi Nejad V Sun H K Hosseini H Adeli SH Wang T. Prevalence of respiratory symptoms and spirometric changes among non-smoker male wood workers. PLoS One. (2020) 15:e0224860. doi: 10.1371/journal.pone.0224860, PMID: 32187180 PMC7080227

[77] Anyfantis ID Rachiotis G Hadjichristodoulou C Gourgoulianis KI. Respiratory symptoms and lung function among Greek cotton industry workers: A cross-sectional study. Int J Occupat Environ Med. (2017) 8:32–8. doi: 10.15171/ijoem.2017.888, PMID: 28051194 PMC6679635

[78] Meo SA. Dose responses of years of exposure on lung functions in flour mill workers. J Occup Health. (2004) 46:187–91. doi: 10.1539/joh.46.187, PMID: 15215659

