# Supplementary material for: The effect of occupational exposure to organic dust on lung function parameters among African industrial workers: a systematic review and meta-analysis
Source: Front Public Health. 2024 Nov 1;12:1424315. doi: 10.3389/fpubh.2024.1424315 (PMC11563806; doi:10.3389/fpubh.2024.1424315)
Supplement: Supplementary file 1 [file Table_1.DOCX]

| Author and publication year | Evaluation criteria | | | Total score |
| --- | --- | --- | --- | --- |
|  | **Selection** | **Comparability** | **Outcome** |  |
| Abaya et al. 2019 | ******** | ****** | ****** | 8 |
| Abaya et al, 2018 | ******* | ****** | ****** | 7 |
| Abdulsalam Saliu Tosho, 2015 | ******** | ****** | ****** | 8 |
| A. J. Ugheoke et al, 2006 | ******* | ****** | ****** | 7 |
| Nigisti Abraha, 2014 | ******* | ***** | ****** | 6 |
| Adeoye, et al,2014 | ******* | ***** | ****** | 6 |
| Asgedom et al. 2019 | ******** | ****** | ****** | 8 |
| Demeke and Haile, 2018 | ******** | ****** | ****** | 8 |
| Derso et al, 2021 | ******* | ****** | ****** | 7 |
| Dunga JA, et al,2015 | ******** | ****** | ******* | 9 |
| Ennin et al, 2017 | ******** | ****** | ******* | 9 |
| Fahim and El-Prince, 2013 | ******* | ***** | ****** | 6 |
| Fentie et al., 2019 | ******* | ****** | ****** | 7 |
| H.A. Mohammadien et al., 2013 | ******** | ****** | ******* | 9 |
| Hamed O. Khalifa, 2003 | ******* | ***** | ****** | 6 |
| Hinson et al. 2014 | ******** | ****** | ****** | 8 |
| Hinson et al. 2016 | ******** | ****** | ******* | 9 |
| Ibekwe and Okojie 2014 | ******** | ****** | ******* | 9 |
| Ige and Awoyemi 2002 | ******** | ***** | ****** | 7 |
| Ijadunola et al, 2005 | ******** | ****** | ***** | 7 |
| Jabur et al, 2022 | ******** | ****** | ****** | 8 |
| K. Iyogun et al.,2019 | *** | ** | ** | 7 |
| Kanko et al, 2017 | ******* | ****** | ****** | 7 |
| Lagiso et al., 2020 | ******** | ****** | ****** | 8 |
| Mwelange et al. 2019 | ******** | ****** | ******* | 9 |
| Obem Okwari, 2005 | ******** | ****** | ******* | 9 |
| Omigie et al, 2023 | ******** | ***** | ****** | 7 |
| Omole oj et al., 2018 | ******** | ****** | ****** | 8 |
| Sakwari et al. 2013 | ******** | ****** | ******* | 9 |
| Tobin et al. 2015 | ******** | ****** | ******* | 9 |
| Ulanga et al, 2021 | ******* | ***** | ****** | 6 |
| Virginia Kimanzi, 2022 | *** | ** | ** | 7 |

**Table 1:** The Newcastle-Ottawa Quality Assessment of articles included in this systematic review and meta-analysis.
